# Positive Role of Purpose in Life in Health Outcomes and Perspectives on Environment

**DOI:** 10.1093/geroni/igaa057.1919

**Published:** 2020-12-16

**Authors:** Shyuan Ching Tan, Alyssa Gamaldo, Angela Sardina

**Affiliations:** 1 Penn State University, State College, Pennsylvania, United States; 2 Penn State, University Park, Pennsylvania, United States; 3 University of North Carolina Wilmington, Wilmington, North Carolina, United States

## Abstract

Having a sense of purpose directs behaviors, hence, purpose in life (PIL) can be a useful indicator/moderator of healthy mental and physical behaviors and outcomes. As such, purpose in life, particularly in lower income older adults, might encourage meaningful engagement in activities and life that lead to positive health. Thirty-nine residents (M=68.01, SD=10.26) of affordable housing for older adults in Wilmington, NC and State College, PA were surveyed on demographics, mental health, well-being (i.e., PIL), health behaviors, and their perceptions on immediate housing and the community resources. Findings suggest that for higher educated, younger and Black older adults, PIL moderates or protects against negative mental outcomes (p<.05). For higher educated older adults, PIL moderates or encourages positive perception of job opportunities in the community (p<.05) and healthier behaviors (p<.05). More research is needed to understand how environment interacts with PIL to promote healthy behaviors and outcomes.

